# Surface-Modified Pore-Filled Anion-Exchange Membranes for Efficient Energy Harvesting via Reverse Electrodialysis

**DOI:** 10.3390/membranes13120894

**Published:** 2023-11-30

**Authors:** Ji-Hyeon Lee, Do-Hyeong Kim, Moon-Sung Kang

**Affiliations:** Department of Green Chemical Engineering, College of Engineering, Sangmyung University, Cheonan 31066, Republic of Korea; jh980106jj@naver.com (J.-H.L.); dohyeongkim665@gmail.com (D.-H.K.)

**Keywords:** pore-filled anion-exchange membranes, polypyrrole, reduced graphene oxide, reverse electrodialysis, monovalent ion selectivity, uphill transport

## Abstract

In this study, novel pore-filled anion-exchange membranes (PFAEMs) modified with polypyrrole (PPy) and reduced graphene oxide (rGO) were developed to improve the energy harvesting performance of reverse electrodialysis (RED). The surface-modified PFAEMs were fabricated by varying the contents of PPy and rGO through simple spin coating and chemical/thermal treatments. It was confirmed that the PPy and PPy/rGO layers introduced on the membrane surface did not significantly increase the electrical resistance of the membrane and could effectively control surface characteristics, such as structural tightness, hydrophilicity, and electrostatic repulsion. The PPy/rGO-modified PFAEM showed excellent monovalent ion selectivity, more than four times higher than that of the commercial membrane (AMX, Astom Corp., Tokyo, Japan). This means that the PPy/rGO layer can effectively reduce the permeation of multivalent ions with a high charge intensity and a relatively large hydration radius compared to monovalent ions. The results of evaluating the performance of the surface-modified PFAEMs by applying them to a RED cell revealed that the decrease in potential difference occurring in the membrane was reduced by effectively suppressing the uphill transport of multivalent ions. Consequently, the PPy/rGO-modified membrane exhibited a 5.43% higher power density than the AMX membrane.

## 1. Introduction

Recently, as climate change accelerates, interest in eco-friendly renewable energy technologies with low or no carbon emissions is increasing around the world. Salinity gradient energy (SGE), one of the promising renewable energy technologies, is a clean energy source that produces electricity via the difference in chemical potential between two solutions with different salt concentrations without emitting pollutants [1,2].

Reverse electrodialysis (RED), one of the representative salinity difference power generation technologies using membranes, uses the concentration difference between seawater and river water as a driving force to selectively move ions through an ion-exchange membrane (IEM) and can generate electric power via the redox reactions that occur in the electrode compartments. In the RED process, efficient power generation is possible because chemical energy caused by the concentration difference in the solutions is directly converted into electrical energy [3,4,5].

The RED stack is composed of a series of anion-exchange membranes (AEMs) and cation-exchange membranes (CEMs) arranged alternately between two electrodes, and the IEMs included in the stack are separated by a gasket (spacer) to allow an electrolyte solution to flow. Cations and anions move in opposite directions through cross-arranged IEMs, and at this time, a potential difference occurs within the stack. The electrode solution containing redox couples circulates between the two electrode compartments [5]. An oxidation-reduction reaction occurs on the electrode surfaces at both ends of the stack, and the electrons generated move through an external circuit connecting both electrodes, generating electricity [6].

In the RED process, IEMs are a critical component in determining the stack performance. The high permselectivity and low electrical resistance of IEMs are direct influencing factors that can improve the power generation performance of RED [7]. Additionally, for successful RED applications, IEMs must simultaneously have excellent durability and be inexpensive. Some commercial IEMs contain woven or nonwoven fabric support to enhance the mechanical properties. For this reason, the thickness of the IEM increases, increasing the membrane resistance and the production cost due to the complex manufacturing process [8,9]. Instead of these expensive commercial IEMs, developing IEMs with low resistance and high permselectivity remains a challenge.

Among various types of IEMs, pore-filled IEMs (PFIEMs), which are fabricated through thermal- or photo-polymerization by filling the pores of highly porous support with a prepolymer solution, are believed to be promising for improving the power density of RED. Due to the characteristics of the porous substrate used, the PFIEM has the advantage of having excellent mechanical strength despite a relatively thin film thickness, resulting in low electrical resistance. In addition, it has the feature of suppressing the excessive swelling of the membrane caused by the hydration of the ion exchange groups and maintaining high ion exchange capacity (IEC) [10]. Therefore, recently, many studies on PFIEMs for high-performance RED stacks have been reported. For example, Choi et al. fabricated an anion-exchange membrane (AEM) with an approximate thickness of 25 µm by filling a porous support with an electrolyte based on vinyl benzyl trimethylammonium chloride (VBTMA) and dopamine methacrylamide with a catechol group. The fabricated membrane with an optimal condition was shown to have excellent ion selectivity and low electrical resistance, resulting in 18% higher performance than the RED stack employing a combination of Neosepta CMX and AMX membranes [11]. In addition, Kim et al. used a 27 µm-thick porous substrate to prepare PFAEMs and PFCEMs by filling the pores with *N*,*N*-bis(acryloyl)piperazine and (vinylbenzyl)trimethylammonium chloride, and *N*,*N*′-ethylenebis(acrylamide) and vinyl sulphonic acid, respectively. A power density of up to 2.4 W/m^2^ was obtained from the RED test with a combination of the prepared PFAEM and PFCEM [12]. Several research results have also shown that IEMs fabricated through the pore-filling method effectively enhance RED performance, owing to low electrical resistance and excellent mechanical properties [13,14,15].

Meanwhile, not only monovalent ions but also multivalent ions (i.e., Mg^2+^, Ca^2+^, and SO_4_^2−^) exist in seawater and river water flowing into RED. When a feed solution containing such multivalent ions is used, the selective permeability of monovalent ions through the membranes could be reduced. That is, undesirable ion exchange could occur between multivalent ions in a low-concentration solution and monovalent ions in a high-concentration solution due to a concentration gradient. This uphill transport phenomenon increases membrane resistance, causing a decrease in the open-circuit voltage (OCV) and power density of REDs [16,17]. Therefore, it is desirable to use a monovalent ion-selective IEM to prevent the penetration of multivalent ions through the membrane for efficient REDs. The monovalent ion selective separation mechanisms of IEM could be explained based on size exclusion and electrostatic repulsion [18]. First, effective size exclusion can be achieved by increasing the degree of cross-linking, forming a dense layer on the membrane surface, and elevating the surface hydrophobicity through chemical modifications with light irradiation or heat treatment [19,20,21]. For example, monovalent ions (e.g., Cl^−^, 317 kJ/mol) have a much lower value of Gibbs hydration energy than divalent ions (e.g., SO_4_^2−^, 1000 kJ/mol). Accordingly, IEMs with a hydrophobic surface can allow more monovalent ions with a relatively small hydration radius to pass through. In addition, the monovalent selectivity could be enhanced by adjusting the relative magnitude of electrostatic repulsion [22].

Meanwhile, polypyrrole (PPy) can be easily synthesized through the electrochemical polymerization or chemical oxidation polymerization of pyrrole (Py) and has the advantage of producing a compound with high yield at low cost [23]. Until recently, studies have been conducted to control the selectivity for specific ions using PPy as a modifying material for IEMs. For example, Salmeron-Sanchez et al. immersed commercial CEM and AEM in a solution of Py and H_2_SO_4_ and then immersed them in FeCl_3_ solution, an oxidizing agent, to oxidize Py and polymerize it into PPy [24]. The modified IEMs significantly reduced the crossover of viologen derivatives (BP7) and TEMPOL, which are active organic molecules, in aqueous organic redox flow batteries (AORFB) and improved the battery performance [24]. In addition, Tufa et al. developed PPy-chitosan composite materials with different concentrations of Py (0.025–1 M) at a variety of polymerization times (0–8 h) and used them to modify CEM to prevent the transport of multivalent ions for enhancing the RED performance. As a result, the OCV and power density were improved by 20% and 42%, respectively, compared to the initial performance before the membrane modification [25]. In this way, PPy is believed to be one of the best materials for forming a very dense structure on the membrane surface and increasing electrostatic repulsion against multivalent ions due to its chemical structure that can be partially charged [25].

Moreover, graphene oxide (GO), an oxidized form of graphene, is a two-dimensional nanomaterial that contains abundant oxygen-containing functional groups, such as epoxy, hydroxyl, and carboxyl. GO has been widely used for fabricating various separation membranes because it has excellent electrical conductivity, high mechanical strength, a large specific surface area, etc. [26]. Generally, GO can be chemically reduced by using capping reagents (hydrogen sulfide, hydrazine, sodium borohydride, dimethylhydrazine, hydroquinone, etc.), resulting in reduced GO (rGO). rGO has low solubility in organic solvents and a strong π-π stacking tendency, forming aggregates irreversibly [27,28,29,30,31,32]. Amarnath et al. reported the reduction of a GO using PPy as a reducing agent for the first time. According to the proposed mechanism, Py is first oxidized to release electrons, and GO is reduced using these electrons. As a result, GO reduced through this process revealed to form graphene sheets with PPy [27]. In addition, Yang et al. reported that the formation mechanism of a GO-PPy composite with a lamellar nanostructure is related to hydrogen bonding, electrostatic interactions, and π-π stacking between PPy and GO [33].

As mentioned above, PPy and rGO are believed to be effective materials that could be used to improve the performance of separation membranes. Therefore, in this study, surface-modified composite AEMs were developed by introducing a PPy/rGO layer on the base membrane to improve the performance of RED. A pore-filled AEM (PFAEM) was fabricated by employing a porous polyethylene film as a substrate and filling the pores with an ionomer. After that, a thin coating layer with various PPy/rGO ratios was introduced on the base membrane surface. We attempted to optimize the monovalent ion selectivity of the membranes by adjusting the hydrophobicity and charge density of the membrane surface according to the ratio of PPy and rGO. The electrochemical properties of surface-modified composite AEMs were systematically analyzed using various methods, and a surface modification composition optimized for the RED process was derived by evaluating the monovalent ion selectivity. Finally, the composite PFAEM prepared under optimal conditions was applied to a RED cell to confirm its performance.

## 2. Materials and Methods

### 2.1. Preparation of the PFAEM

**Figure 1:**
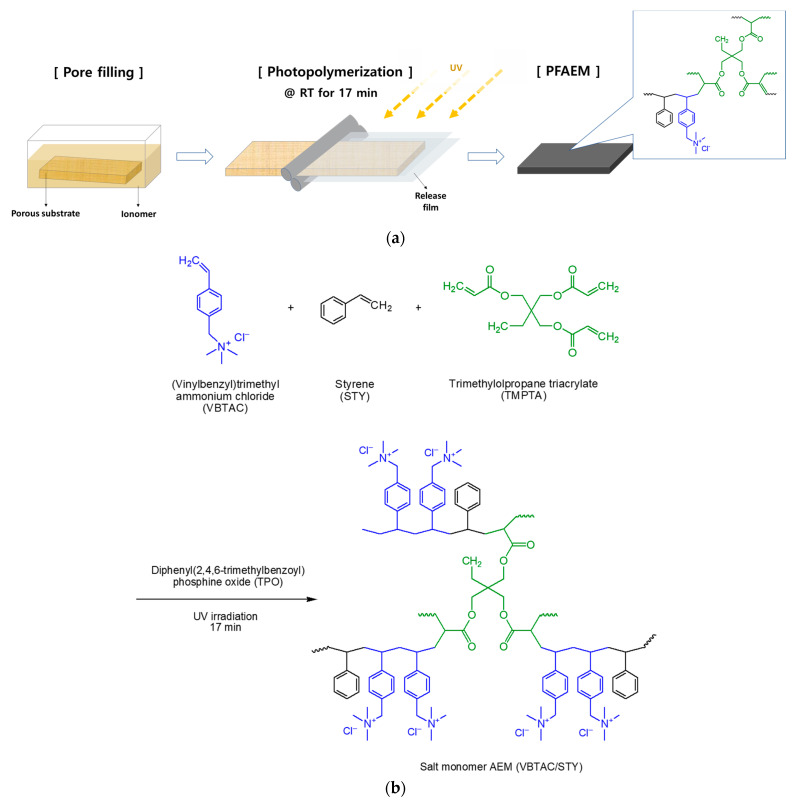
(**a**) Preparation procedure of the PFAEM and (**b**) reaction schemes for the anion-exchange polymer.

In this study, a porous film (Hipore, thickness = 25 μm, Asahi Kasei E-materials Co., Tokyo, Japan) composed of polyethylene (PE), which is used as a separator for the secondary batteries, was used as a substrate to prepare the PFAEMs. (Vinylbenzyl)trimethylammonium chloride (VBTAC) and styrene (STY) were selected as monomers to prepare the base AEM. In addition, trimethylolpropane triacrylate (TMPTA) was used as a cross-linking agent, and diphenyl(2,4,6-trimethylbenzoyl)phosphine oxide) (TPO) was chosen as a photoinitiator. Ethanol (EtOH) was used as a solvent that could dissolve all the components and was added at a weight ratio of 1/3 compared to the VBTAC monomer. The molar ratio of VBTAC and STY monomers was fixed at 1:1, and the contents of the cross-linker and photoinitiator were determined to be 10 wt% and 5 wt%, respectively, based on the total monomer weight. The porous substrate was then immersed in the prepared monomer mixture for 1 h. When the pores of the substrate were filled with monomer, it was inserted between the release films, rolled to remove excess monomer present on the film surface, and then cured using a UV lamp (TL-K 40W/10R, Philips, Amsterdam, The Netherlands) at room temperature for 5 min. The fabrication process of the pore-filled base membrane and the synthesis process of the anion-exchange polymer are shown in Figure 1. When the polymerization was completed, the release film was removed from the pore-filled membrane and stored in 0.5 M NaCl solution. All reagents used for the base membrane preparation were purchased from Sigma-Aldrich (St. Louis, MO, USA) and used without further purification.

### 2.2. Surface Modifications of the PFAEM

**Figure 2:**
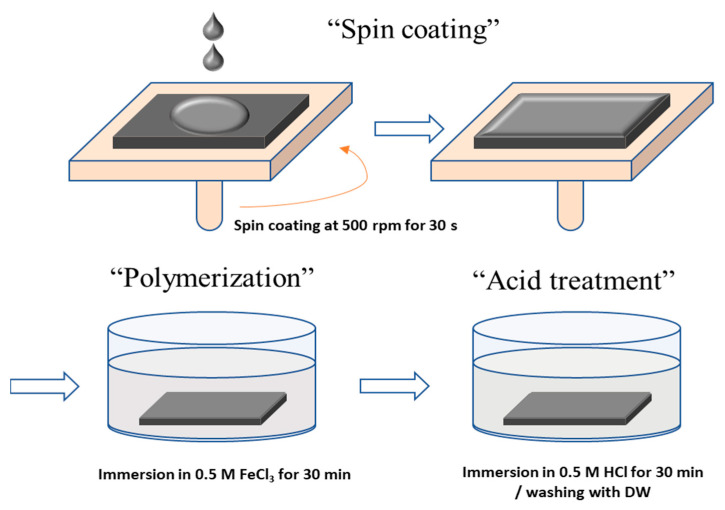
Procedure of the membrane surface modification with PPy using a spin coating method.

First, surface modification was performed by coating a Py solution on the surface of the prepared PFAEM. A spin coating method was used to modify the membrane surface, and the spin coating was carried out at 500 rpm for 30 s. At this time, the Py solution was prepared as 5 wt% using an acetonitrile (ACN) solvent. The Py-coated membrane was immersed in a 0.5 M FeCl_3_ aqueous solution to undergo polymerization for 30 min and then treated in 0.5 M HCl for 30 min. After the surface modification was completed, the membrane was washed with distilled water and then stored in 0.5 M NaCl. All reagents used for the surface modification of PPy were purchased from Sigma-Aldrich (St. Louis, MO, USA) and used without further purification. The process of introducing the PPy layer onto the basement membrane surface is illustrated in Figure 2.

**Figure 3:**
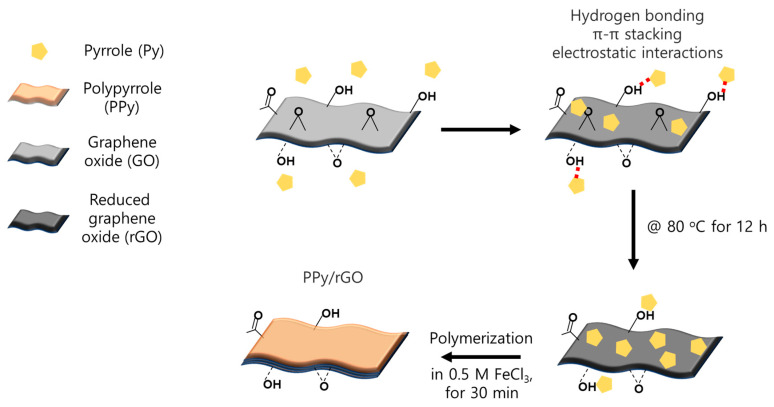
Schematic illustration of the preparation procedure for the PPy/rGO composite layer.

Next, for the membrane surface modification using PPy/rGO, a coating solution was prepared by adding GO to the Py solution. The GO content was varied as 1~5 wt% in 5 wt% Py solution. The coating solution was heated at 80 °C for 12 h to reduce GO, and sonication was carried out for 30 min to finally prepare a Py-rGO coating solution. GO could be reduced to rGO through two steps and is explained as follows. First, Py is oxidized and becomes an oligomer, and then it is reduced to rGO through a chemical reduction mechanism in which GO receives the electrons released from Py and is reduced [23,29]. At this stage, the Py oligomer and rGO are bound to each other through hydrogen bonds, π-π stacking, and electrostatic interactions. Afterward, the reduction of GO is completed via heat treatment at 80 °C for 12 h. PPy/rGO was coated on the base membrane using the same spin-coating method as PPy and then immersed in a 0.5 M FeCl_3_ aqueous solution to proceed with the polymerization reaction for 30 min. It was treated in 0.5 M HCl for 3 h, washed with DW, and finally stored in 0.5 M NaCl. The preparation procedure of the PPy-rGO composite layer is shown in Figure 3.

### 2.3. Membrane Characterization

**Figure 4:**
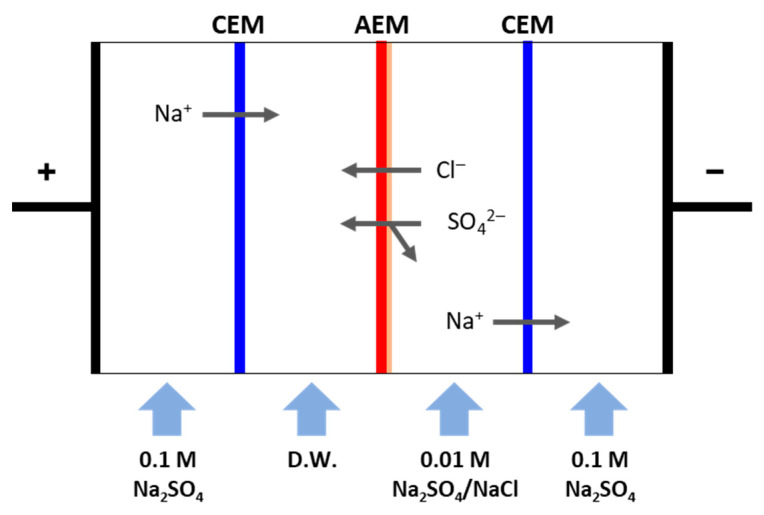
Schematic drawing of the electrodialysis experiment for measuring the monovalent anion selectivity of the membranes.

The morphological characteristics of the surface and cross-section of the prepared PFAEMs were observed using a field emission scanning electron microscope (FE-SEM, MIRA LMH, TESCAN, Brno, Czech Republic). Additionally, to analyze the chemical structure of the prepared membranes, the absorption spectra were measured using Fourier transform infrared spectroscopy (FT-IR, FT/IR-4700, Jasco, Tokyo, Japan). The surface hydrophilicity of the membranes was evaluated using a contact angle analyzer (Phoenix 150, SEO Co., Suwon, Republic of Korea). The crystallinity of GO, PPy, and PPy/rGO was also confirmed through X-ray diffraction (XRD, SmartLab, Rigaku Co., Tokyo, Japan) analysis. Meanwhile, the interelement bonding of the surface modification layer was analyzed using an X-ray photoelectron spectrometer (XPS, K-Alpha, Thermo Fisher Scientific Inc., Waltham, MA, USA). Additionally, to investigate the electrostatic properties of the surface, the zeta potential of the membranes was measured in the pH range of 2 to 11 using an electrokinetic analyzer (SurPASS 3, Anton Paar GmbH, Graz, Austria). The electrical resistance (ER) of the membranes was measured in 0.5 M NaCl using a lab-produced 2-point probe clip cell and an impedance analyzer (SP-150, Bio-Logic Science Instruments, Seyssinet-Pariset, France), and the values were calculated using the following equation [34]:

(1) $$\mathrm{ER}=\left({\left|Z\right|}_{sample}\cdot \cos\theta_{sample}-{\left|Z\right|}_{blank}\cdot \cos\theta_{blank}\right)\times A$$

where ${\left|Z\right|}_{sample}$ is the impedance of the electrolyte and membrane (Ω), ${\left|Z\right|}_{blank}$ is the impedance of the electrolyte only (Ω), *θ* is the phase angle (degree), and *A* is the membrane area (cm^2^). The transport number for the anion (*t^−^*), which indicates the anion-selective permeability, was measured using the emf method with a 2-compartment diffusion cell and was calculated using the following equation:

(2) $$E_{m}=\frac{RT}{F}(1-2t^{-})\ln\frac{C_{L}}{C_{H}}$$

where *E_m_* is the measured cell potential, *R* is the gas constant, *T* is the absolute temperature, *F* is the Faraday constant, and *C_L_* and *C_H_* are the concentrations of the NaCl solution, which were 1 mM and 5 mM, respectively. At this time, the cell potential was measured by placing a pair of Ag/AgCl electrodes in both compartments and connecting them to a digital voltmeter. The chronopotentiometry could be usefully used to study the transport phenomena of ions through IEMs [35]. In this study, the chronopotentiometric curves of the membranes were measured using a 2-compartment cell with an Ag/AgCl reference electrode and a pair of Ag/AgCl plates connected to a potentiostat/galvanostat (SP-150, Bio-Logic Science Instruments, France) at a constant current density of 4.46 mA/cm^2^ for 100 s. The transition time (*τ*) determined from the chronopotentiometric curve was substituted into the modified Sand equation (Equation (3)) to determine the ratio (*ε*) of the conductive area on the surface of IEM [36].

(3) $$\varepsilon =\frac{2i\tau^{1/2}\left(t_{}^{m}-t_{}^{b}\right)}{C_{}^{b}z_{}F{\left(\pi D\right)}^{1/2}}$$

where *i* is the current density, *z* is the valence of counter ion, *F* is the Faraday constant, *D* is the diffusion coefficient, *t^m^* and *t^b^* are the transport numbers in the membrane and solution, respectively, and *C^b^* is the concentration of the NaCl solution (0.025 M). The monovalent ion selectivity of the membranes was determined through a lab-produced 4-compartment cell experiment. A pair of platinum-coated titanium plates were used as the electrodes, and 0.1 M Na_2_SO_4_ was used as the electrode solution. The effective area of the membrane was 15 cm^2^, and 0.01 M NaCl/Na_2_SO_4_ was used as the dilute compartment solution and distilled water (DW) was used as the concentrate compartment solution. During the measurement, the surface-modified layer of the PFAEM was positioned toward the dilute compartment. The monovalent ion selectivity experiment was carried out for 1 h under a constant current condition of 15 A/m^2^. The concentrations of Cl^−^ and SO_4_^2−^ ions in the concentration compartment were determined using ion chromatography (883 Basic IC plus, Metrohm, Herisau, Switzerland). The ion flux was obtained from the analyzed ion concentrations and substituted into Equation (4) to determine the monovalent ion selectivity ($P_{{SO}_{4}^{2-}}^{{Cl}^{-}}$) [37,38,39,40].

(4) $$P_{{\mathrm{SO}}_{4}^{2-}}^{{\mathrm{Cl}}^{-}}=\frac{J_{{\mathrm{Cl}}^{-}}\cdot C_{{\mathrm{SO}}_{4}^{2-}}}{J_{{\mathrm{SO}}_{4}^{2-}}\cdot C_{{\mathrm{Cl}}^{-}}}$$

where $J_{{Cl}^{-}}$ and $J_{{SO}_{4}^{2-}}$ are the fluxes (mol/cm^2^·s) of Cl^−^ and SO_4_^2−^ ions, respectively, and $C_{{Cl}^{-}}$ and $C_{{SO}_{4}^{2-}}$ are the concentrations (mol/L) of SO_4_^2−^ and Cl^−^ ions, respectively, sampled in the concentration compartment. The cell configuration and principle of the 4-compartment cell used to measure the monovalent ion selectivity of the membranes are illustrated in Figure 4.

### 2.4. RED Performance

**Figure 5:**
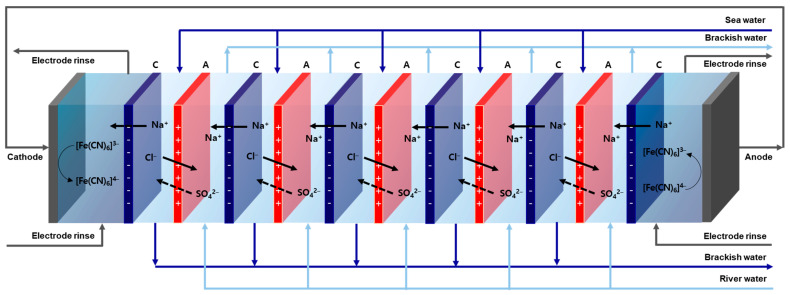
Schematic representation of the RED stack consisting of 5 cell pairs (C: CEM; A: AEM).

The RED performance test was performed in the galvanostatic mode by connecting a lab-produced RED stack to a potentiostat/galvanostat (SP-150, Bio-Logic Science Instruments, France), and the system configuration is shown in Figure 5. The effective area of the electrodes and IEMs was 15 cm^2^, and two Pt-coated Ti plates were used as the electrodes. The electrode compartment solution was prepared by dissolving a redox couple of 0.05 M K_4_Fe(CN)_6_/0.05 M K_3_Fe(CN)_6_ in a 0.25 M Na_2_SO_4_ aqueous solution. The composition of seawater and river water was 0.459 M NaCl + 0.051 M Na_2_SO_4_ and 0.0153 M NaCl + 0.0017 M Na_2_SO_4_, respectively, and the solutions were supplied at 50 mL/min. During the RED experiments, the surface-modified layer of the PFAEMs was positioned toward the river water compartment. The RED stack consisted of 5 cell pairs and was tested by varying the current density from 0 to 14 A/m^2^.

## 3. Results and Discussion

**Figure 6:**
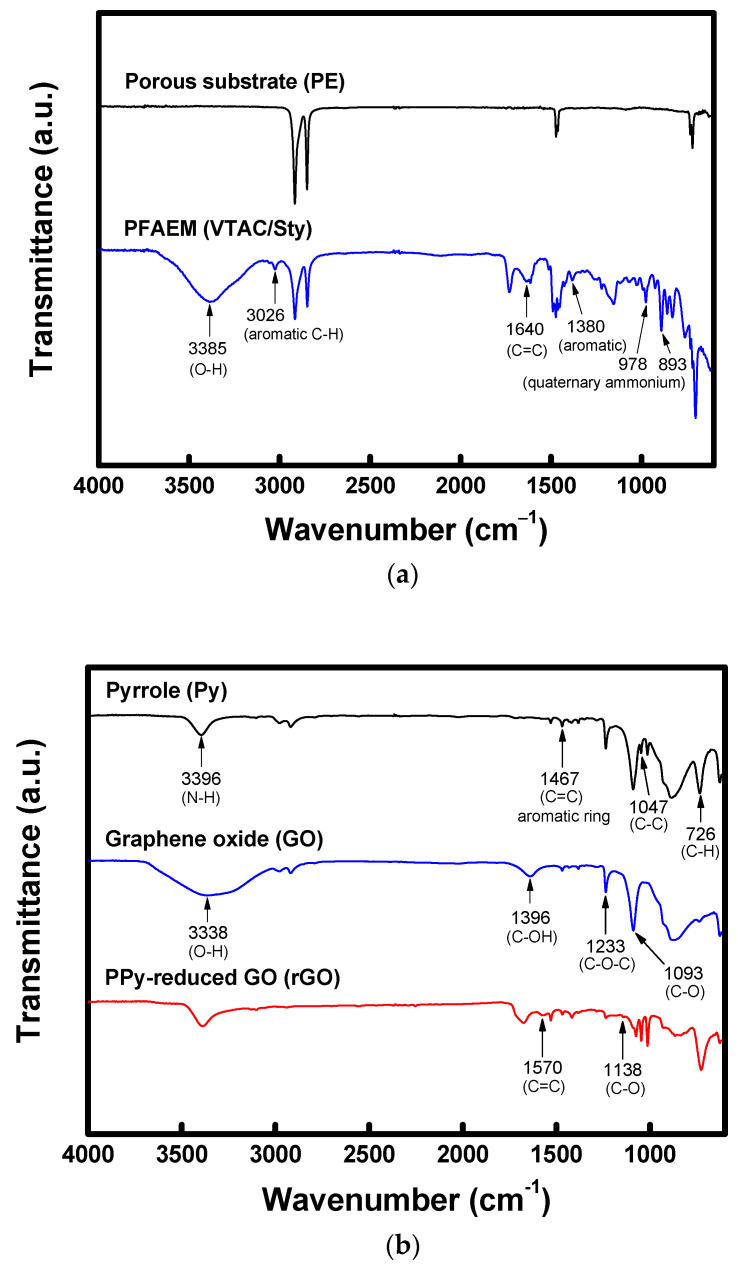
FT-IR spectra of the (**a**) porous substrate (PE) and PFAEM (VBTAC/STY); (**b**) surface modification layer.

The FT-IR spectra of the porous support and prepared PFAEM are shown in Figure 6a. The spectrum of the PFAEM is significantly different from that of the PE support, and an O-H stretching vibration was confirmed at 3385 cm^−1^, which indicates the introduction of hydrophilic ion exchange groups into the polymer [41]. Meanwhile, an aromatic C-H bond was observed from the absorption band at 3026 cm^−1^ [42], and a C=C bond and aromatic ring by VBTAC and STY were confirmed from the absorption bands observed at 1640 and 1380 cm^−1^, respectively [43,44]. The existence of the quaternary ammonium group bound to VBTAC was also confirmed by the absorption bands of 978 and 893 cm^−1^ [45,46]. Therefore, it could be confirmed that the anion-exchange polymer was well combined with the porous PE substrate.

Meanwhile, Figure 6b shows the FT-IR spectra of Py, GO, and PPy/rGO used for the surface modification. The vibration at 3396 cm^−1^ observed in the spectrum of Py is due to the in-plane stretching of N-H. Additionally, 1467, 1047, and 726 cm^−1^ represent the absorption peaks for C=C, C-C, and C-H contained in Py, respectively. In the spectrum of GO, a broad stretching band of an O-H group was shown around 3338 cm^−1^. In addition, other peaks corresponding to the stretching modes of C-OH, C-O-C, and C-O were observed at 1396, 1233, and 1093 cm^−1^, respectively. In the spectrum of PPy/rGO, where the thermal reduction reaction was completed, it was confirmed that the broad O-H stretching band observed in the spectrum of GO disappeared, and only the N-H peak of PPy was observed. The C-OH absorption band at 1396 cm^−1^ disappeared and the epoxy ring peaks at 1233 and 1093 cm^−1^ also decreased. These results demonstrate that GO was successfully reduced to rGO through Py polymerization and the thermal reaction [45,46].

**Figure 7:**
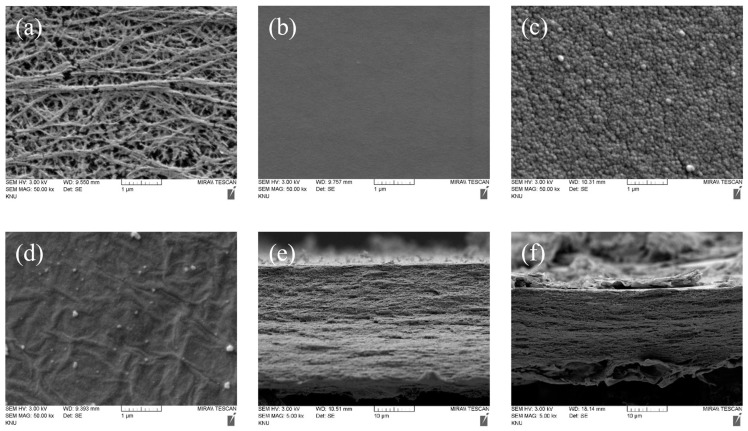
FE-SEM images of the surface of the (**a**) porous substrate, (**b**) PFAEM, (**c**) PPy-modified PFAEM, (**d**) PPy/rGO-modified PFAEM; the cross-section of (**e**) PPy-modified PFAEM and (**f**) PPy/rGO-modified PFAEM.

The FE-SEM images for the morphological observation of the surface and cross-section of the porous substrate and prepared membranes are shown in Figure 7. Figure 7a reveals the surface of the porous PE substrate used to fabricate the membrane, and as shown in Figure 7b, the pores of the porous substrate were completely filled with the polymer, resulting in a smooth surface without defects. In the case of the membrane coated with a PPy layer, it can be seen from Figure 7c that the surface roughness largely increased due to the existence of PPy grains. As shown in Figure 7d, the membrane incorporating the PPy/rGO layer showed a greatly reduced roughness compared to the PPy layer and a typical wrinkled surface morphology due to rGO [47]. Figure 7e,f displays the cross-sectional images of the PPy- and PPy/rGO-modified membranes, respectively. The thickness of the PPy-coated layer was confirmed to be approximately 150–200 nm, and the PPy/rGO layer was thought to be somewhat thicker than the PPy layer.

**Figure 8:**
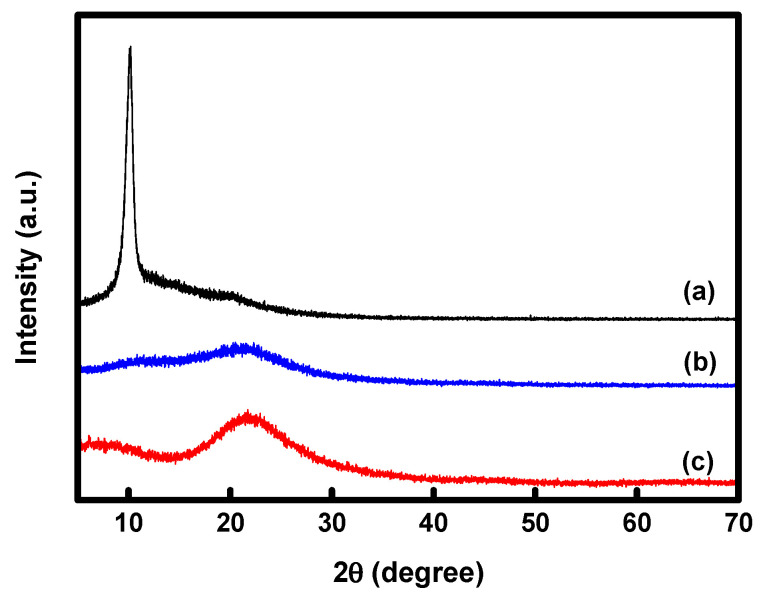
XRD patterns of the (**a**) GO, (**b**) PPy, and (**c**) PPy/rGO composites.

The XRD spectra of GO, PPy, and PPy/rGO are shown in Figure 8. The intrinsic diffraction peak of GO was confirmed at 2*θ* = 10.5° in the GO spectrum. Additionally, a broad peak was identified around 2*θ* = 21.6° in the PPy spectrum, representing the amorphous characteristics of PPy. In the spectrum of PPy/rGO, the sharp peak of GO observed at 2*θ* = 10.5° disappeared, and instead, a broad peak was observed around 2*θ* = 21.8°. This is similar to the peak identified from the spectra of PPy/rGO reported in the literature, demonstrating that GO was converted to rGO and PPy and rGO were completely integrated [48,49].

**Figure 9:**
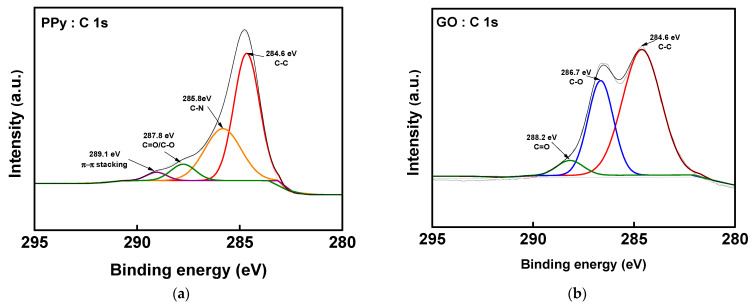

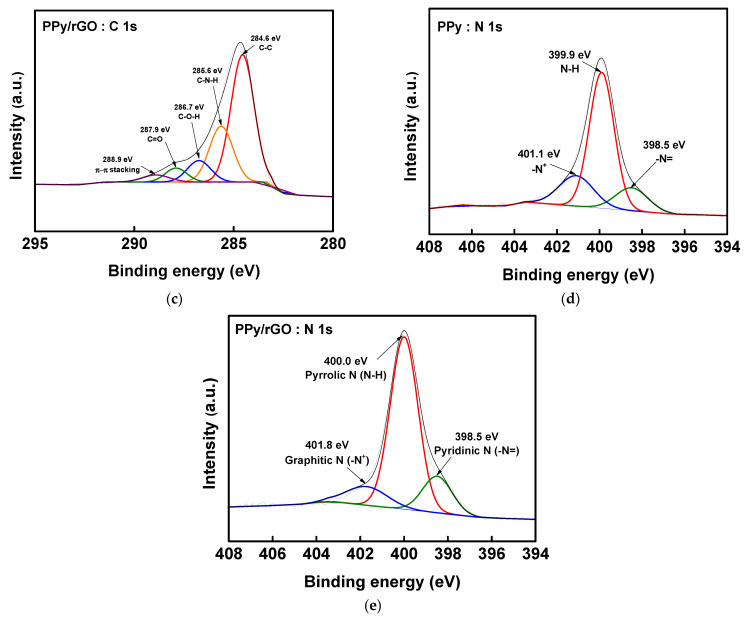
XPS spectra of C 1s of (**a**) PPy, (**b**) GO, and (**c**) PPy/rGO; N 1s of (**d**) PPy and (**e**) PPy/rGO.

Figure 9a–c shows the C 1s spectra of the PPy, GO, and PPy/rGO composites, respectively. π-π stacking is a non-covalent interaction that occurs between pi bonds of aromatic rings and can occur between aromatic moieties of “PPy and PPy” or “PPy and rGO”, and this interaction can be confirmed through XPS analysis [50,51]. From the PPy spectrum in Figure 9), a peak indicating π-π stacking between the PPy molecules was confirmed at 289.1 eV [50]. Moreover, by comparing this to Figure 9b,c, it can be seen that the intensity of the C-O and C=O peaks appearing in the XPS spectrum of GO was greatly reduced in PPy/rGO, indicating the reduction of GO [52]. It could also be confirmed that PPy and rGO were combined through the C-N peak that appears at 285.6 eV in the PPy/rGO spectrum [50,51,52,53,54]. The peak assigned to π-π stacking that appears at 288.9 eV in the spectrum of PPy/rGO indicates that the π-π interaction occurs between the PPy molecules or between PPy and rGO [52,55]. Figure 9d,e shows the deconvoluted N 1s spectra of PPy and PPy/rGO, respectively. The N 1s spectrum of PPy could be separated into the -N= peak of the imine nitrogen appearing at 398.5 eV, the N-H peak of the neutral amine nitrogen appearing at 399.9 eV, and the -N^+^ peak of the positively charged nitrogen appearing at 401.1 eV. In addition, from the spectrum of PPy/rGO, the peaks corresponding to a pyridinic nitrogen (-N=, at 398.5 eV), a pyrrolic nitrogen (N-H, at 400.0 eV), and a graphitic nitrogen (-N^+^, at 401.8 eV) were observed, demonstrating that PPy was bound to rGO [50,51,52,53,54]. Meanwhile, in the N 1s XPS spectrum of PPy/rGO, all peaks shifted to a larger binding energy compared to PPy. This is believed to be mainly a result of π-π stacking and hydrogen bonding between rGO and PPy [52].

**Figure 10:**
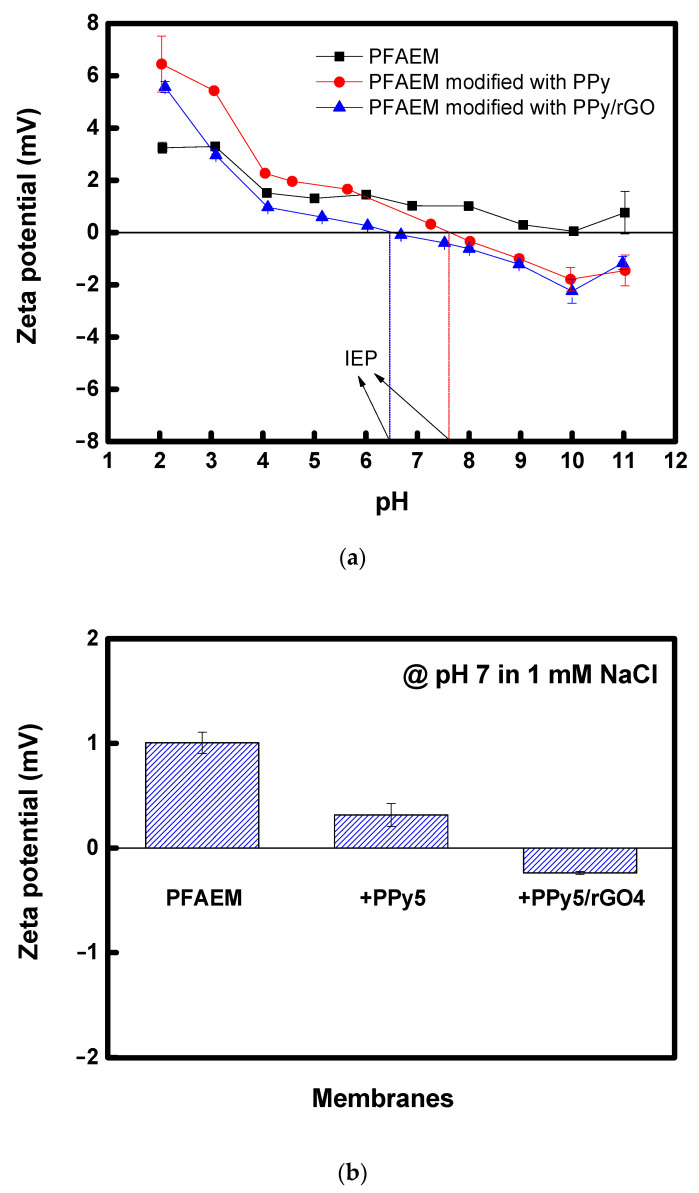
Zeta potential values of the prepared PFAEMs: (**a**) Zeta potentials according to the solution’s pH (the vertical dotted lines represent the IEPs.); (**b**) Zeta potentials at pH 7.

The surface charge characteristics have a significant impact on the properties of monovalent ion-selective membranes. Therefore, the zeta potential of the prepared membranes was measured, and the results are shown in Figure 10. The zeta potential was measured in the pH range of 2 to 11, and all membranes exhibited positive zeta potential values below a pH of 6.45. This is because all the membranes were positively charged due to the quaternary ammonium groups. In the cases of the membranes modified with PPy, they could also be positively charged due to the protonation of the pyrrole moiety under acidic conditions. However, as the pH of the solution increases, the deprotonation of PPy progresses, and the zeta potential of the membrane gradually becomes negative through the ionization of the functional groups (e.g., -COOH and -OH) contained in rGO. As a result, for the membranes modified with PPy and PPy/rGO, the zeta potential of the membrane became zero, and the isoelectric point (IEP), where the polarity changes, was observed. The IEP of the PPy-modified membrane was approximately 7.60, and that of the PPy/rGO-modified membrane was revealed to be 6.45. As shown in Figure 10b, based on a pH of 7, the relative negative charge increased in the order of PFAEM < PPy-modified PFAEM < PPy/rGO-modified PFAEM. Because these surface negative charges could increase the electrostatic repulsion against multivalent anions, it could be expected that the monovalent ion selectivity of the membrane modified with PPy/rGO, which shows the highest negative surface potential, would be the best among the prepared membranes.

**Figure 11:**
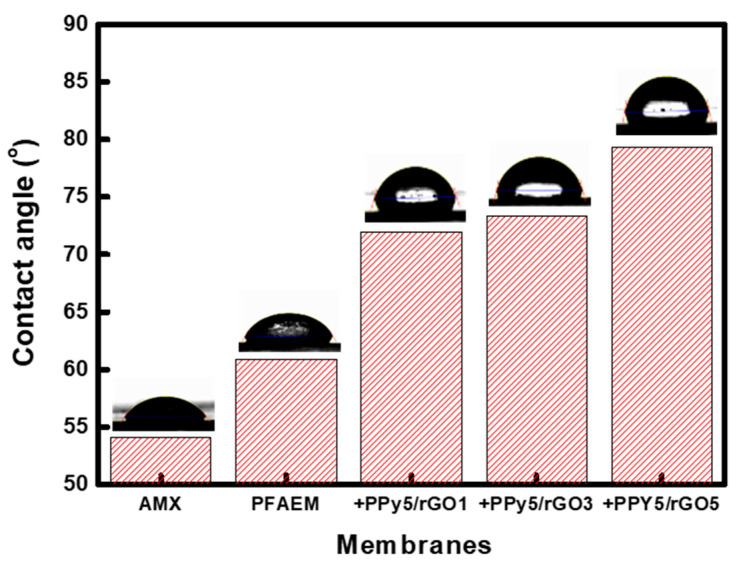
Water contact angles of AMX, PFAEM, and PPy/rGO-modified PFAEMs.

Meanwhile, the hydrophilicity of the membrane surface is a factor that significantly affects the ion transport characteristics through the membrane. In this study, the contact angle was measured to check the change in hydrophilicity of the membrane surface through surface modification, and the results are displayed in Figure 11. Among the samples tested, a commercial membrane (AMX, Astom Corp., Tokyo, Japan) showed the smallest contact angle (54°), meaning that it had the highest surface hydrophilicity. In comparison, the PFAEM showed a somewhat higher contact angle of approximately 61°. Since the surface of the PFAEM was completely covered by the ionomer, as shown in Figure 7b, this contact angle represents the intrinsic hydrophilicity of the pore-filled ionomer. With the introduction of the PPy/rGO layer on the membrane surface, the contact angle increased significantly, which was due to an increase in the density of the membrane surface and a decrease in the number of ion exchange groups. Moreover, as the content ratio of hydrophobic rGO increased, the contact angle increased, indicating that rGO had a significant effect on increasing the hydrophobicity of the membrane surface. The increased hydrophobicity by introducing the PPy/rGO layer could allow for a denser surface structure, increasing the sieving effect for multivalent anions with a relatively large hydration radius.

**Figure 12:**
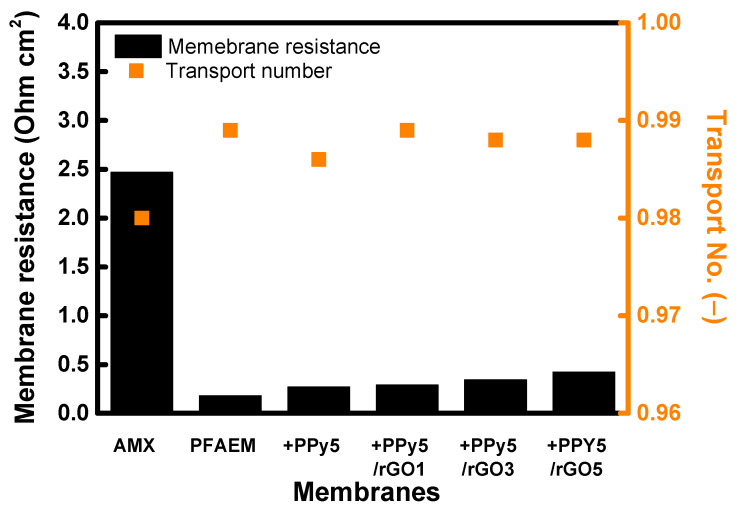
Membrane resistance and transport number values of AMX, PFAEM, PPy-modified PFAEM, and PPy/rGO-modified PFAEMs.

The values of the electrical resistance and transport number of the PFAEM and the PFAEMs modified with PPy and PPy/rGO compared with those of the AMX membrane are shown in Figure 12. In particular, PPy/rGO modification was performed with various PPy and rGO content ratios, and the sample name was indicated as PPyX/rGOY. Here, X and Y refer to the weight fraction of PPy and rGO, respectively. As a result, the PFAEM and PFAEMs modified with PPy and PPy/rGO showed significantly lower electrical resistance than AMX, which was due to the relatively thin membrane thickness (note that thicknesses of AMX and PFAEM were 140 μm and 25 μm, respectively). However, it was confirmed that the membrane resistance slightly increased as the PPy layer was coated. This could be interpreted as the surface being densely modified due to the introduction of the PPy layer. Additionally, in the case of the PPy/rGO-modified membrane, the membrane resistance tended to increase slightly as the rGO content increased, meaning that the transfer of ions through the membrane was hindered by the increase in surface hydrophobicity and structural tightness caused by introducing rGO. However, since the modified layer’s thickness was sufficiently thin, the effect of the resistance of the modified layer on the overall membrane resistance was not significant. Meanwhile, the ion transport number for the counter ion (Cl^−^) for all the membranes tested was above 0.98, and the ion transport numbers for the prepared membranes were somewhat higher than that for the commercial membrane. As a result, it could be confirmed that through modification using PPy and PPy/rGO, the surface properties of the PFAEM could be effectively controlled without deteriorating the overall membrane resistance and ion transport properties.

**Figure 13:**
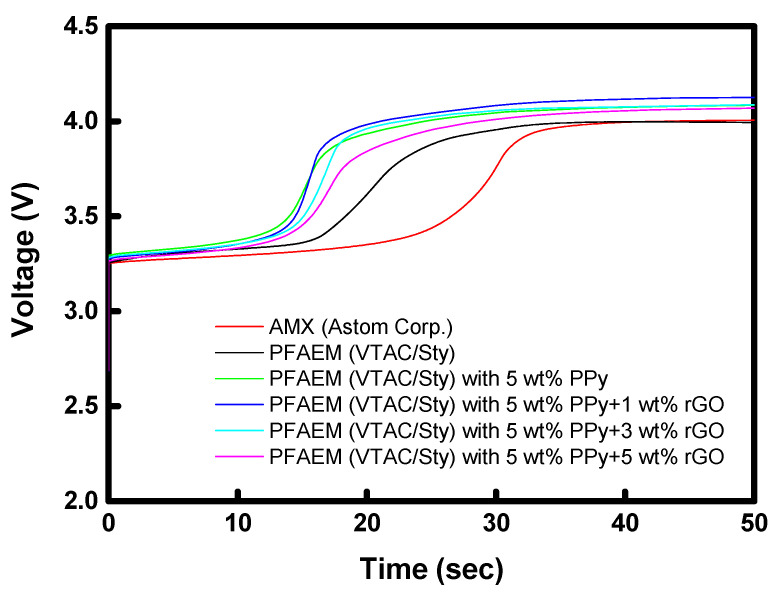
Chronopotentiometric curves of AMX, unmodified PFAEM, PPy-modified PFAEM, and PPy/rGO-modified PFAEMs.

Figure 13 shows the chronopotentiometric curves of AMX, unmodified PFAEM, PPy-modified PFAEM, and PPy/rGO-modified PFAEMs. In this test, a constant current was applied to the membrane and the time-course change in membrane potential was measured. From this, the transition time (*τ*), during which the membrane potential changes rapidly, was determined, and the fraction of the conductive region (*ε*) on the membrane surface was calculated by substituting it into the modified Sand equation (Equation (3)) [36]. The *ε* values of the membrane surface determined are also summarized in Table 1. As a result, AMX, a homogeneous IEM, revealed the largest *ε* value among the membrane samples tested. However, in the case of PFAEM, which is a base membrane, the *ε* value was relatively small compared to AMX, which is thought to be a result of reflecting the area of inert PE used as a support when fabricating the membrane. In addition, with the introduction of the PPy layer on the PFAEM surface, the density of the fixed charge groups on the surface decreased, resulting in a significant decrease in the *ε* value. However, as the ratio of rGO in the PPy/rGO layer increased, the *ε* values were slightly increased, which is believed to be owing to the influence of the ionic functional groups (-COOH and -OH) contained in rGO. From these results, it could be confirmed that the electrostatic charge strength of the membrane surface could be effectively adjusted through modification with PPy and PPy/rGO.

**Figure 14:**
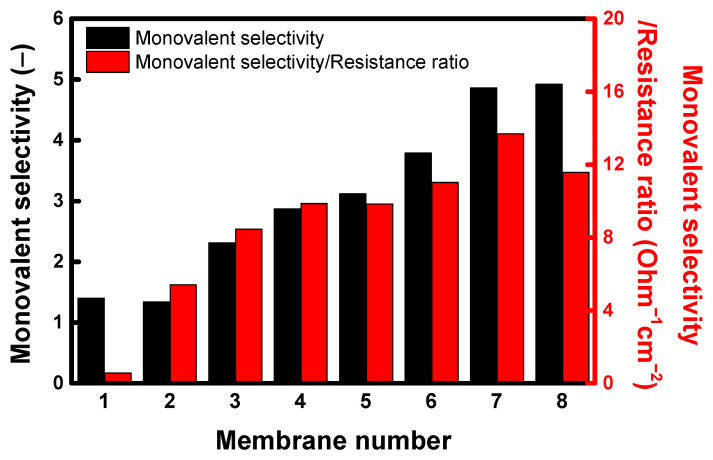
Monovalent selectivity of chlorine ions against sulfate ions of different membranes and monovalent selectivity/membrane resistance ratios (membrane number: 1. AMX, 2. unmodified PFAEM, 3. PPy5-, 4. PPy5/rGO1-, 5. PPy5/rGO2-, 6. PPy5/rGO3-, 7. PPy5/rGO4-, and 8. PPy5/rGO5-modified PFAEMs).

Figure 14 exhibits the monovalent selectivity of the chlorine ions against the sulfate ions of different membranes and the monovalent selectivity/membrane resistance ratios. It was observed that AMX and unmodified PFAEM have low monovalent ion selectivity of 1.4 and 1.32, which are close to 1, respectively. However, the monovalent ion selectivity was significantly improved as the PPy and PPy/rGO layers were introduced to the membrane surface. In particular, the monovalent ion selectivity of the membrane tended to increase in proportion to the rGO content up to 5 wt%. These results mean that the electrostatic repulsion and sieving exclusion effect for the divalent ions on the membrane surface increased as the PPy and PPy/rGO layers were introduced. That is, multivalent anions have a greater repulsive force with the negative charge on the membrane surface than monovalent ions and also possess a large hydration radius (e.g., 0.332 nm for Cl^−^ and 0.379 nm for SO_4_^2−^), rendering it relatively difficult to pass through the highly dense and hydrophobic PPy and PPy/rGO layers [56,57,58]. In addition, we attempted to derive the optimal surface modification conditions for the membrane by calculating the ratio between monovalent ion selectivity and membrane resistance. Since monovalent ion selectivity and membrane resistance have a trade-off relationship, membrane resistance elevates as monovalent ion selectivity increases. Therefore, the ratio of monovalent ion selectivity to membrane resistance was highest at 4 wt% rGO and tended to decrease after 5 wt% rGO, where the increase in monovalent ion selectivity slowed down. As a result, the PPy5/rGO4 (PPy 5 wt%/rGO 4 wt%) condition was determined as the optimal membrane modification condition considering both the monovalent ion selectivity and membrane resistance.

**Figure 15:**
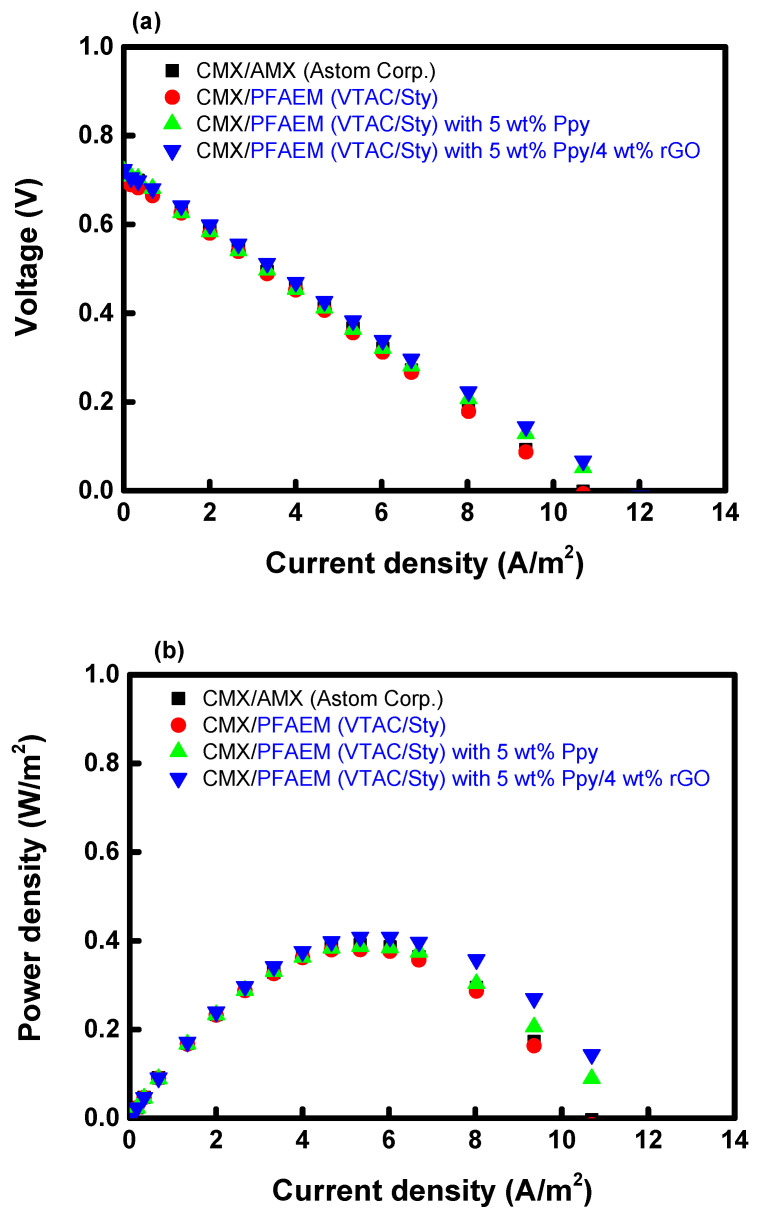
(**a**) Current-voltage and (**b**) current-power density curves of RED cells employing AMX, unmodified PFAEM, PPy-modified PFAEM, and PPy/rGO-modified PFAEMs, respectively.

Uphill transport occurring in RED refers to the phenomenon in which multivalent ions present in river water exchange with monovalent ions in seawater against the concentration gradient, which reduces the OCV and power density of RED [59]. In this study, to reduce the uphill transport, a PFAEM with excellent monovalent ion selectivity was fabricated through PPy/rGO modification. The RED performance evaluation results employing different AEMs are summarized in Figure 15 and Table 2. As a result, it was shown that the OCV of unmodified PFAEM was slightly lower than that of AMX but was improved through surface modification with PPy and PPy/rGO. This is believed to be because the PPy and PPy/rGO layers effectively suppressed the uphill transport of multivalent ions, thereby reducing the decrease in potential difference occurring in the membrane. These results led to an increase in power density, and the PPy- and PPy/rGO-modified membranes showed that the power densities increased by 3.17% and 7.65%, respectively, compared to the base membrane. Moreover, it was confirmed that both the PPy- and PPy/rGO-modified PFAEMs showed higher power densities compared to the AMX membrane. It may be thought that the effect of improving the RED performance using this PPy/rGO-modified membrane is not sufficient. However, since this is a result measured in a lab-produced cell with a few cell pairs and a small effective area, it is expected that a significantly higher power density improvement would be achieved when used in a practical RED stack.

## 4. Conclusions

In this study, we successfully developed high-performance PFAEMs modified with PPy and rGO for the RED application. The PFAEM was fabricated by filling pores of a porous PE support with a salt monomer and a cross-linking agent and successively performing in situ photopolymerization. The surface of the PFAEM was then modified with PPy and rGO through spin-coating and successive chemical and thermal treatments. In particular, the membrane surface characteristics, such as structural tightness, hydrophilicity, and electrostatic repulsion, were shown to be effectively controlled by controlling the contents of PPy and rGO. The monovalent ion selectivity was measured using a mixed solution of monovalent and divalent ions, and the monovalent ion selectivity was more than four times higher than that of the commercial AMX membrane. Moreover, the optimal surface modification condition was derived by considering the ratio of monovalent ion selectivity and membrane resistance in the trade-off relationship between them. The PPy- and PPy/rGO-modified PFAEMs were applied to a RED cell to effectively suppress the uphill transport of multivalent ions, thereby reducing the decrease in potential difference occurring in the membrane. These results led to an increase in power density, and the PPy- and PPy/rGO-modified PFAEMs showed that the power densities increased by 1.03% and 5.43%, respectively, compared to the commercial membrane. Recently, the monovalent ion selectivity for IEMs is required not only in RED but also in various electro-membrane processes. The results of this study are expected to provide valuable information for developing IEMs with excellent monovalent ion selectivity required in a variety of electro-membrane processes.

## Figures and Tables

**Table 1:** Fraction of the conductive region of AMX, unmodified PFAEM, PPy-modified PFAEM, and PPy/rGO-modified PFAEMs.

Membrane	*ε* (−)
AMX (Astom Corp.)	0.993
PFAEM	0.802
PFAEM + 5 wt% PPy	0.719
PFAEM + 5 wt% PPy/1 wt% rGO	0.741
PFAEM + 5 wt% PPy/3 wt% rGO	0.750
PFAEM + 5 wt% PPy/5 wt% rGO	0.754

**Table 2:** OCV values and power density of RED cells employing AMX, unmodified PFAEM, PPy-modified PFAEM, and PPy/rGO-modified PFAEMs, respectively.

Membranes	OCV (V)	Power Density (W/m^2^/Cell Pair)
CMX/AMX	0.712	0.387
CMX/PFAEM	0.708	0.379
CMX/PFAEM + 5 wt% PPy	0.720	0.391
CMX/PFAEM + 5 wt% PPy/4 wt% rGO	0.724	0.408

## Data Availability

Data is contained within the article.
